# Across Generations: A University Course Promoting Intergenerational Relationships

**DOI:** 10.1093/geroni/igaa057.018

**Published:** 2020-12-16

**Authors:** Megan Foti

**Affiliations:** Stockton University, Galloway, New Jersey, United States

## Abstract

In order to support University students in meeting general studies requirements, while also promoting knowledge and understanding related to aging, Across Generations was developed. Across Generations provides students with a basic understanding of intergenerational relationships and the importance of fostering intergenerational collaborations in order to positively influence our communities. Beyond topics in older adulthood, this course helps students learn more about different generations, diverse cultures, and how to interact and form meaningful relationships with those different from them. Students also have an opportunity to interact with older adults and reflect on their experiences. Additionally, they complete a needs assessment and program development activities in order to identify relevant needs and identify ways to address them. In some cases, actual programs are developed and continue after the course ends. Across Generations topics include: introduction to aging and intergenerational relationships, learning from each other and the benefits of fostering intergenerational relationships, public policy, grandfamilies, older adult isolation, cross-cultural considerations, conflict resolution from an older adult viewpoint, and identifying needs and creating opportunities. This presentation shares the course development process, course learning outcomes and topic outline, student experiences and feedback, and recommendations for similar course development.

